# Continuity of Genetic Risk for Aggressive Behavior Across the Life-Course

**DOI:** 10.1007/s10519-021-10076-6

**Published:** 2021-08-14

**Authors:** Camiel M. van der Laan, José J. Morosoli-García, Steve G. A. van de Weijer, Lucía Colodro-Conde, the ACTION Consortium, Hill F. Ip, Camiel M. van der Laan, Eva M. L. Krapohl, Isabell Brikell, Cristina Sánchez-Mora, Ilja M. Nolte, Beate St Pourcain, Koen Bolhuis, Teemu Palviainen, Hadi Zafarmand, Lucía Colodro-Conde, Scott Gordon, Tetyana Zayats, Fazil Aliev, Chang Jiang, Carol A. Wang, Gretchen Saunders, Ville Karhunen, Anke R. Hammerschlag, Daniel E. Adkins, Richard Border, Roseann E. Peterson, Joseph A. Prinz, Elisabeth Thiering, Ilkka Seppälä, Natàlia Vilor-Tejedor, Tarunveer S. Ahluwalia, Felix R. Day, Jouke-Jan Hottenga, Andrea G. Allegrini, Kaili Rimfeld, Qi Chen, Yi Lu, Joanna Martin, María Soler Artigas, Paula Rovira, Rosa Bosch, Gemma Español, Josep Antoni Ramos Quiroga, Alexander Neumann, Judith Ensink, Katrina Grasby, José J. Morosoli, Xiaoran Tong, Shelby Marrington, Christel Middeldorp, James G. Scott, Anna Vinkhuyzen, Andrey A. Shabalin, Robin Corley, Luke M. Evans, Karen Sugden, Silvia Alemany, Lærke Sass, Rebecca Vinding, Kate Ruth, Jess Tyrrell, Erik A. Ehli, Fiona A. Hagenbeek, Eveline De Zeeuw, Toos C. E. M. Van Beijsterveldt, Henrik Larsson, Harold Snieder, Frank C. Verhulst, Najaf Amin, Alyce M. Whipp, Tellervo Korhonen, Eero Vuoksimaa, Richard J. Rose, André G. Uitterlinden, Andrew C. Heath, Pamela Madden, Jan Haavik, Jennifer R. Harris, Øyvind Helgeland, Stefan Johansson, Gun Peggy S. Knudsen, Pal Rasmus Njolstad, Qing Lu, Alina Rodriguez, Anjali K. Henders, Abdullah Mamun, Jackob M. Najman, Sandy Brown, Christian Hopfer, Kenneth Krauter, Chandra Reynolds, Andrew Smolen, Michael Stallings, Sally Wadsworth, Tamara L. Wall, Judy L. Silberg, Allison Miller, Liisa Keltikangas-Järvinen, Christian Hakulinen, Laura Pulkki-Råback, Alexandra Havdahl, Per Magnus, Olli T. Raitakari, John R. B. Perry, Sabrina Llop, Maria-Jose Lopez-Espinosa, Klaus Bønnelykke, Hans Bisgaard, Jordi Sunyer, Terho Lehtimäki, Louise Arseneault, Marie Standl, Joachim Heinrich, Joseph Boden, John Pearson, LJohn Horwood, Martin Kennedy, Richie Poulton, Lindon J. Eaves, Hermine H. Maes, John Hewitt, William E. Copeland, Elizabeth J. Costello, Gail M. Williams, Naomi Wray, Marjo-Riitta Järvelin, Matt McGue, William Iacono, Avshalom Caspi, Terrie E. Moffitt, Andrew Whitehouse, Craig E. Pennell, Kelly L. Klump, S. Alexandra Burt, Danielle M. Dick, Ted Reichborn-Kjennerud, Nicholas G. Martin, Sarah E. Medland, Tanja Vrijkotte, Jaakko Kaprio, Henning Tiemeier, George Davey Smith, Catharina A. Hartman, Albertine J. Oldehinkel, Miquel Casas, Marta Ribasés, Paul Lichtenstein, Sebastian Lundström, Robert Plomin, Meike Bartels, Michel G. Nivard, Dorret I. Boomsma, Michelle K. Lupton, Brittany L. Mitchell, Kerrie McAloney, Richard Parker, Jane M. Burns, Ian B. Hickie, René Pool, Jouke-Jan Hottenga, Nicholas G. Martin, Sarah E. Medland, Michel G. Nivard, Dorret I. Boomsma

**Affiliations:** 1 Biological Psychology, Vrije Universiteit, Van der Boechorststraat 7, 1081 BT Amsterdam, The Netherlands; 2 The Netherlands Institute for the Study of Crime and Law Enforcement, Amsterdam, The Netherlands; 3 QIMR Berghofer Medical Research Institute, Brisbane, QLD Australia; 4 Faculty of Health Sciences, The University of Sydney, Sydney, Australia; 5 Brain and Mind Centre, University of Sydney, Camperdown, Australia

**Keywords:** Aggressive behavior, Aggression, Life-course, Development, Polygenic score, Rolling weights

## Abstract

We test whether genetic influences that explain individual differences in aggression in early life also explain individual differences across the life-course. In two cohorts from The Netherlands (*N* = 13,471) and Australia (*N* = 5628), polygenic scores (PGSs) were computed based on a genome-wide meta-analysis of childhood/adolescence aggression. In a novel analytic approach, we ran a mixed effects model for each age (Netherlands: 12–70 years, Australia: 16–73 years), with observations at the focus age weighted as 1, and decaying weights for ages further away. We call this approach a ‘rolling weights’ model. In The Netherlands, the estimated effect of the PGS was relatively similar from age 12 to age 41, and decreased from age 41–70. In Australia, there was a peak in the effect of the PGS around age 40 years. These results are a first indication from a molecular genetics perspective that genetic influences on aggressive behavior that are expressed in childhood continue to play a role later in life.

**Supplementary Information:**

The online version contains supplementary material available at 10.1007/s10519-021-10076-6.

## Introduction

Aggression is broadly defined as common human behavior that intends to cause harm, by verbal, psychological, and physical means, to others (Baron and Richardson 1994; Anderson and Bushman 2002). Physical aggression tends to peak at age 2–4 years and then decreases (Alink et al. 2006; Cairns et al. 1989; Cairns and Cairns 1994; Karriker-Jaffe et al. 2008; Loeber and Stouthamer-Loeber 1998; Tremblay et al 2004; Tremblay 2010), as neurological, cognitive and social development empower children with other means to get what they want. Social or relational aggression emerges in the preschool years, continues through childhood and adolescence and subsequently declines in adulthood (e.g. Underwood 2003).

The relative positions in terms of aggression (i.e. rank order) in the population persist across the life-course (Pulkkinen and Pitkänen 1993; Tuvblad and Baker 2011). In other words, the most aggressive child often grows up to be the most aggressive adult (Farrington 1989). There has been some debate about the continuation of individual differences in aggression from childhood to adulthood. Moffitt (1993) argued that this statistical continuation is driven by a small number of highly aggressive individuals in a population who remain aggressive throughout their lives, the ‘life-course persistent’ individuals. The rest, she argues, are the ‘adolescent limited’ type, for whom aggressive behavior is limited to adolescence. Although it is clear that the ‘life-course persistent’ individuals explain part of the stability in aggression, Huesmann et al. (2009) showed that most individuals retain their relative position in a population, regardless of their starting position. Several factors have been identified that help explain individual differences in continuity of aggression, such as parenting, peers, socioeconomic and cultural context, mental processes and genetic predisposition (Boomsma 2015; Farrington 1989; Labella and Masten 2018; Murray and Farrington 2010; Tolan et al. 2013; Vuoksimaa et al. 2021).

Twin and family studies, mostly focusing on children, indicate that genetic factors explain around 50% of the variation in aggression (Veroude et al. 2016). Across the lifespan, heritability estimates of aggression and antisocial behavior seem to increase somewhat from childhood through adulthood, as the importance of shared environmental effects decreases (Tuvblad and Baker 2011; Waltes et al. 2016; Odintsova et al. 2019). Although individuals retain their genetic make-up throughout their lives, this does not necessarily imply that the same genetic variants play a role in aggression across the life-course. Studies with longitudinal twin designs show that genetic factors contribute significantly to the stability of aggression during preschool age, school age, and puberty (van Bijsterveldt et al. 2003; Porsch et al. 2016). These results led us to test the hypothesis that genetic variants that are expressed on aggression during childhood and adolescence also are significantly associated with aggression later in the life-course.

Odintsova and colleagues (2019) published an extensive overview of the current state of genomics aggression research, concluding that clear genome wide significant effects have not yet been found in genetic association studies (GWAS). This is partly attributable to the fact that aggression, like many other complex human behaviors, is influenced by a multitude of individual genetic variants, each of which likely has a small effect. From this ‘polygenic’ genetic architecture, arises the need for very large GWAS sample sizes. Ip and colleagues (2021) conducted a genome wide meta-analysis (GWAMA) of aggression phenotypes in children and adolescents, aged 3 to 18 years. In a GWAMA, results from GWAS in multiple cohorts are combined with the aim to increase statistical power to find associations between a genetic marker (usually a single nucleotide polymorphism, i.e. SNP) and an outcome (phenotype). If phenotypes and genetic effects are comparable across cohorts from different ages and backgrounds, small effects of SNPs that do not attain significance in a single cohort may be genome wide significant in the GWAMA. In the Ip et al. paper (2021), a total of 29 cohorts contributed 163 univariate GWAS to the early life aggression GWAMA. This resulted in a total of 328,935 observations from 87,485 unique individuals, aged 3 to 18 years. Observations were across multiple raters, coming from teachers, parents, and self-reports. The Ip et al. (2021) aggression GWAMA is the largest childhood aggression GWAS to date, but no single genome-wide significant hits were observed. Despite this lack of single significant hits, Ip et al. (2021) demonstrated that polygenic scores (PGSs), which sum the effects of a range of genetic markers, with markers included based on whether their *p*-value from the GWAS clears any of 16 thresholds between P = 1 and P < 1.0E-5, explained between 0.036 and 0.44% of the phenotypic variance in aggression in a hold-out sample of 7 year-old Dutch children (*N* = 4491). In an Australian hold-out sample, childhood PGSs explained up to 0.2% of retrospectively assessed childhood conduct disorder. PGSs performed best when markers where included with relatively lenient P-value thresholds, indicating the polygenic nature of aggression phenotypes. Although effect sizes were small, we expect the effect to be large enough to test the hypothesis that there are continuing genetic effects across the life-course.

### The Current Study

In this study, we test the hypothesis that genetic risk factors, measured as DNA variants associated with increased aggressive behavior in early life (Ip et al. 2021), increase the risk of aggression across the life-course. We quantified the contribution of a large number of variants by computing PGSs, and tested their association with aggression in two cohorts from two different countries, namely The Netherlands and Australia. We introduce a novel method to assess differences in genetic influences across the life-course. In this approach we assess the effect of a PGS on aggression at ages 12–70 in The Netherlands, and 16–73 in Australia, by specifying ‘rolling weights’ for age. Within the framework of a linear mixed model, we model the effect of the PGS at each age represented in the data. At each age, we include phenotype information from surrounding ages. The phenotype information is weighted, where weights are centered at the focus age and decay further away from that center. With this method, sample size differences between ages are small, because more information than just the focus age is taken into account. Thereby, we mitigate the risk that sample size differences between ages drive the effects we find. If, for example, there are only few observations at age 25, we can still use information on adjacent ages to imply the effects at age 25. A significant contribution in adults, of PGSs that are based on a discovery study in children and adolescents, would suggest a partially heritable origin of the stability of individual differences in aggression.

## Method

### Participants

**Fig. 1:**
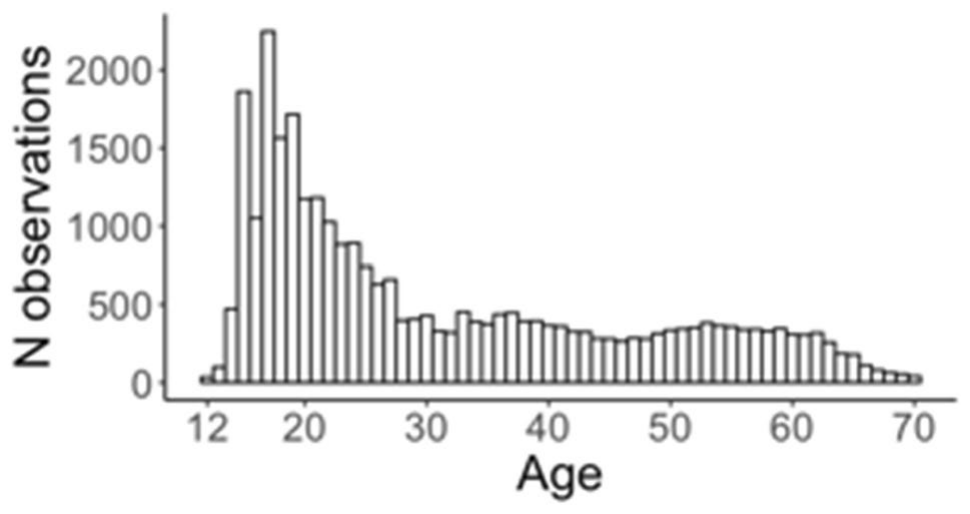
The Netherlands. Age distribution

Dutch participants are registered with The Netherlands Twin Register (NTR; Ligthart et al. 2019). Phenotype data on aggression were collected by survey in six of the data collection waves (1991, 1995, 1997, 2000, 2009, 2014) for twin families who registered as part of the Adult Netherlands Twin Register (ANTR). Twins, whose parents registered them as newborns as part of the Young Netherlands Twin Register (YNTR), and their siblings provided self-ratings of aggression at various ages of the twins. The total phenotyped sample in which aggression scores were computed (Table 1), consisted of families with twins, their siblings, spouses, and parents. In the final analyses only the genotyped individuals could be included. This genotyped sample included 29,454 measures from 13,471 genotyped individuals (62% female) aged 12–70 years (Table 3, Fig. 1). In total, 8705 of these individuals completed more than one questionnaire. All genotyped individuals were from European ancestry, identified based on the top ten 1000-Genomes PCs (Abdellaoui et al. 2013) (Table 2).

**Table 1:** The Netherlands Twin Register

Year	Observations	Mean age (SD)
1991	3325	17.95 (2.24)
1995	3342	19.98 (3.10)
1997	4714	26.73 (10.46)
2000	6684	30.48 (10.75)
2009	14,798	41.44 (15.40)
2014	16,092	40.16 (14.61)
2005–2014 (age twins: 14)	11,080	15.35 (1.54)
2005–2014 (age twins: 16)	8075	17.43 (1.60)
2005–2008 (age twins: 18)	1516	18.88 (1.94)

Collection of aggression data in adolescent and adult twins, their sibs, spouses and parents (1991–2014) and in young twins and their siblings (2005–2014)

This table includes all phenotype observations that were included to calculate IRT aggression scores

**Table 2:** The Netherlands Twin Register: characteristics of genotyped/phenotyped sample

	*N* subjects	*N* measures	Mean age (*SD*)	IRT aggression	
				Mean	*SD*
Total	13,471	29,454	31.35 (15.33)	− 0.02	0.85
Male	5062	10,426	31.34 (16.07)	− 0.07	0.84
Female	8409	19,028	31.35 (14.91)	0.01	0.85

*IRT aggression* item response theory aggression score, *SD* standard deviation

**Table 3:** Australia

Study/year	Observations	Mean age (SD)
16UP (2014–2018)	402	16.34 (0.64)
25UP (2015–2019)	2052	30.05 (4.31)
GHA (2018–2020)	2315	40.45 (14.85)
PISA (2016–2020)	2462	59.98 (6.85)

Data collection (genotyped individuals)

**Fig. 2:**
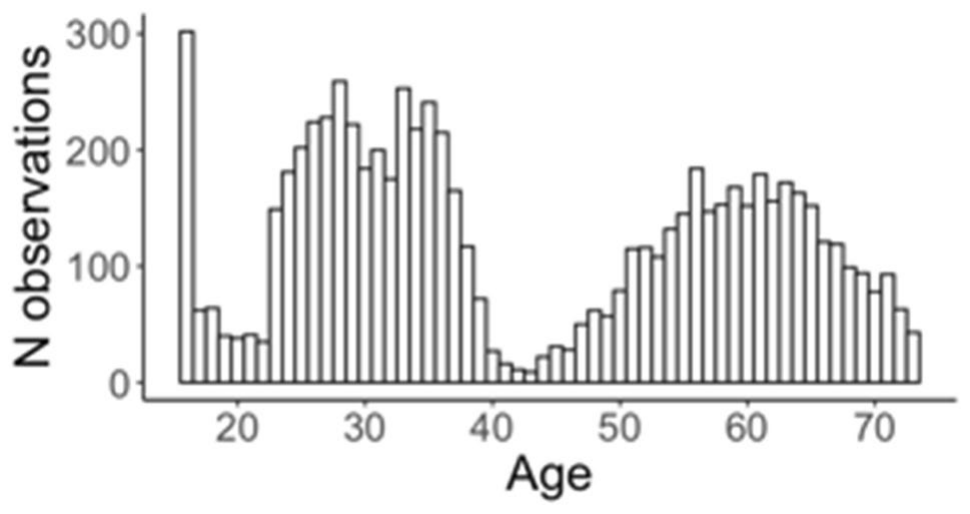
Australia. Age distribution

Australian data came from studies on health and wellbeing collected at QIMR Berghofer Medical Research Institute (QIMRB). A total of 5628 genotyped participants from 2983 families from the Brisbane Longitudinal Twin Study (Wright and Martin 2004), Young and Well (16UP study, Mitchell et al. 2020) and Twenty Five and Up (25UP study, Mitchell et al. 2020) Genetics and Human Agency study (GHA, Morosoli 2020), and Prospective Imaging Study of Ageing (PISA; Lupton et al. 2021) completed surveys which included the Buss-Perry aggression questionnaire (Tables 3 and 4); 1603 people completed the questionnaire twice. At the time of completion, participants were aged 16–73 (Fig. 2).

**Table 4:** Australia

	*N* subjects	*N* measures	Mean age (SD)	IRT aggression	
				Mean	*SD*
Total	5628	7231	42.81 (16.71)	− 0.01	0.95
Male	1904	2385	41.75 (16.84)	0.18	0.90
Female	3724	4846	43.33 (16.62)	− 0.11	0.96

Sample characteristics genotyped individuals

*IRT aggression* item response theory aggression score, *SD* standard deviation

### Phenotyping

#### The Netherlands

**Fig. 3:**
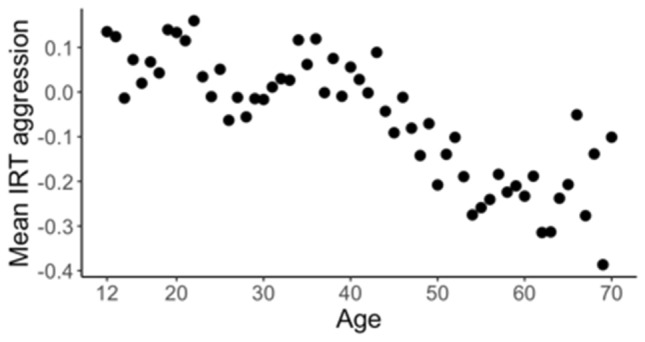
Netherlands. Mean IRT aggression score for each age in the genotyped sample

All participants completed Achenbach System of Empirically Based Assessment self-report questionnaires (ASEBA; Achenbach et al. 2017), either the Youth Self-Report (YSR; Achenbach and Rescorla 2001) or the Adult Self-Report (ASR; Achenbach and Rescorla 2003). In the earlier ANTR surveys, the Young Adult Self Report (YASR) was administered. Surveys in each relevant data collection wave included between 15 and 20 items from the ASEBA aggressive behavior subscale. All items were scored on a three-level scale: 0 = never, 1 = sometimes, 2 = often. Aggression scores were defined separately for each wave of data collection for all NTR participants (i.e. regardless of genotyping status, see Table 1) by Item-Response Theory (IRT; Embretson and Reise 2000) and calculated with the Generalized Partial Credit Model (GPCM) in R (R Core Team 2017), with the mirt package (Chalmers 2012). GPCM is an Item Response Theory model, developed to analyze polytomous data. For each wave of data collection, all participants with a maximum of two missing individual items were included in the GPCM. An IRT-aggression score has benefits over a simple sum-score, because it appropriately weights the relative contributions of individual items to a scale with a more favorable distribution, and takes into account missing data. By fitting a separate model for each wave of data collection, aggression scores for each participant are relative to all other participants in that wave of data collection, thereby filtering out potential ‘wave’ or data collection effects. Because the IRT score for each individual is relative to all other participants in the same wave of data collection, the mean IRT score for each wave is zero. This is reflected in the mean IRT aggression scores at each age (Fig. 3). The final overall IRT aggression score has a mean of 0.00, and ranges from − 1.6 to 4.4, with a standard deviation of 0.86. Only genotyped participants were included for further analysis. The genotyped sample did not differ much from the total sample, with a mean of − 0.02, range from − 1.6 to 3.5, and a standard deviation of 0.84.

#### Australia

**Fig. 4:**
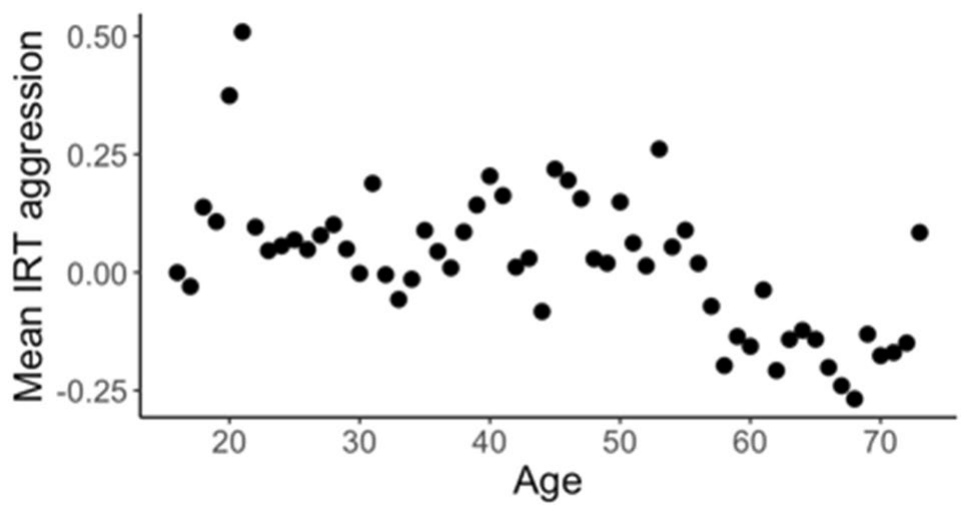
Australia. Mean IRT aggression score for each age

Aggressive behaviour was measured with the Buss–Perry Aggression Questionnaire. This is a 29-item questionnaire in which participants indicate the extent to which statements are characteristic of them (5-point Likert scale, from "extremely uncharacteristic of me" to "extremely characteristic of me", including some items that needed to be reversed). The questionnaire provides a total sum score and four subscores: physical aggression, Verbal aggression, Anger, and Hostility. For 107 out of 7231 observations, missing values on 1 to 6 individual items were imputed using multivariate imputation via the MICE R-package (van Buuren and Groothuis-Oudshoorn 2011). IRT aggression scores were calculated with mirt R-package (Chalmers 2012) within each study (i.e., 16UP, 25UP, GHA, and PISA) for the total aggression score and each of the subscales. Because the IRT score for each individual is relative to all other participants in the same study, the mean score for each study is zero. This is reflected in the mean aggression scores for each age (Fig. 4). The final overall IRT aggression score has a mean of 0.0, and ranges from − 2.9 to 3.8, with a standard deviation of 0.9.

### Genotype Data

The Netherlands: Participants were genotyped on multiple platforms: Affymetrix Axiom, Affymetrix 6.0, Illumina 1 M, Illumina 660, Illumina GSA, Perlegen Affymetrix. Samples with call rate < 0.90, Plink heterozygosity F <  − 0.10 or F > 0.10, and inconsistency of X chromosome genotypes with reported gender were excluded. SNPs with MAF < 1.0E−6, HWE P-value < 1.0E-6, and/or call rate < 0.95 were removed. Genotype data were aligned with the 1000 Genomes reference panel, and filtered for SNPs with allele frequency differences from the CEU population larger than 0.20, palindromic SNPs, and DNA strand issues. DNA Identity By Descent (IBD) state was estimated for all individual pairs using Plink (Purcell et al. 2007) and King (Manichaikul et al. 2010) based on ~ 10.8 k SNPs that all platforms have in common. Samples were removed if IBD did not match expected family relations. CEU population outliers were removed from the data with Smartpca software, based on per platform 1000 Genomes PC projection. Per platform, data were phased using Eagle and imputed to 1000 Genomes with Minimac (Das et al. 2016). The final merged genotype data consist of 12,152,830 SNPs.

Australia: Genotyping was performed on DNA extracted from blood and saliva samples, on Illumina 317 K, 370 K, 610 K, (‘1st generation’), GSA, or Core Exome plus Omni-family (‘2nd generation’) arrays, and GenomeStudio software for genotype calling (Illumina Inc., 200 Lincoln Centre Dr, Foster City, CA 94404). This was followed by imputation from a common SNP set to the 1000 Genomes (Phase 3 Release 5) reference panel, a strategy that allows genotype data from different arrays to be combined. Samples with < 97.5% call rate, non-European ancestry (> 6 SD from the mean European-population cluster for PC1 and PC2) or with familial relationships incompatible with those reported by study participants were excluded. Observed markers were cleaned (by batch) for call rate (≥ 95%); minor allele frequency (≥ 1%); Hardy–Weinberg equilibrium (P ≥ 10^−6^), GenCall score (≥ 0.15 per genotype; mean ≥ 0.7) and standard Illumina filters, before integrating batches and re-running relationship and Mendelian checks. Phasing and imputation were carried out at the Michigan Imputation Server (https://imputationserver.sph.umich.edu/index.html#!) using the 1000 Genomes Phase 3 Release 5 ‘mixed population’ reference panel, with phasing by SHAPEIT followed by imputation using minimac 3 (Das et al. 2016), ‘1st generation’ and ‘2nd generation’ data were imputed separately due to poor overlap between typed markers. Imputation was based on 277,690 (‘1st generation’) and 240,297 (‘2nd generation’) typed markers (passing QC in all relevant batches); and the two were combined after imputation to maximise sample size, using for each individual the ‘1st generation’ imputation if available, otherwise using the ‘second generation’ imputation. This resulted in 9,411,304 SNPs available for analysis, after quality control.

### Polygenic Score Construction

We obtained effect sizes for the association between individual SNPs and aggression from the Ip et al. (2021) GWAMA after omitting the target samples, i.e. analyses were run with no participants from the Netherlands for the Dutch target sample, and no participants from Australia for the Australian target sample (GWAMA sample size for the Netherlands: N_SNPs_ = 7,722,825, N_measures_ = 276,268, N_individuals_ = 81,259, SNP-h^2^ = 3.91%, SE = 0.42. GWAMA sample size for Australia: N_SNPs_ = 7,762,065, N_measures_ = 314,604, N_individuals_ = 75,536, SNP-h^2^ = 3.97%, SE = 0.46). We then computed PGSs for both cohorts with SBayesR V2.03 (Lloyd-Jones et al. 2019), using default settings.

### Statistical Analyses

To ascertain the viability of predicting adult aggression with the PGS that is based on a discovery in 3- to 18-year-olds, we first model the association between the PGS and aggression in the total sample in the Netherlands and in Australia. Here, IRT aggression is predicted from the PGS, age, age^2^, sex, dummy variables for genotyping arrays, and five ancestry-based principal components. We control for the dependence between measures due to relatedness and repeated measures, by adding a random effect for families. Next, we model the effect of the PGS at specific ages. When investigating the effect of PGSs at specific ages, there is a risk that differences in sample size at each age may affect the results. To remedy this, we employ a novel weighted analytic approach in which we make use of more data when looking at specific ages. For each age for which data are available, ages 12 to 70 years in the Dutch context, and ages 16–73 years in the Australian context, we model aggression with the package lme4 (Bates et al. 2015), as a function of the PGS, age, age^2^, sex, dummy variables for genotyping arrays, and five ancestry-based principal components. This means a total of 59 analyses in The Netherlands and 58 in Australia. The models are fitted with weights that weight observations at the focus age as 1 and decay for ages further from that age. In this approach, sample size at each age includes ages around the focus age, but to a lesser extent. In other words, data on surrounding ages are included in the analyses, resulting in larger and more comparable sample sizes at different ages. At each age we control for the dependence between measures due to relatedness and repeated measures, by adding a random effect for families. This captures both dependence due to relatedness and dependence between longitudinal measures. Age covariates are included in the model because a range of ages is still present in each model, albeit with different weights. The mixed effects regression model at each age can be written as:

1 $$Y_{{mif{\text{~}}}} = \beta _{{0f}} {\text{~}} + \beta _{{{\text{Agef}}}} *{\text{Age}}_{{mi}} + \beta _{{1 - {\text{v}}}} *x_{{mi,1 - 13}} + \varepsilon_{{if}}$$

In this notation, Y is the aggression outcome of individual *i* in family *f*, the intercept *β*_*0f*_ is a combination of the population level intercept and the family-level deviation of that intercept, ${{\beta }}_{\mathrm{Agef}}$ is the regression estimate for age and the family-level deviation of that estimate, *Age*_*mi*_ is the age of individual *i* at measure *m*, ${{\beta }}_{1-\mathrm{v}}$ are the regression estimates for all the fixed effects (the PGS, age, age^2^, sex, dummy variables for genotyping arrays, and five ancestry-based principal components.), ${x}_{mi,1-v}$ are the corresponding observed scores of individual *i* at measure *m* for each fixed effect, and ${\epsilon }_{if}$ is the combined individual and group level error term. In matrix notation, this gives:

2 $$Y=X\bullet \beta +Z\bullet b+\epsilon$$

In this notation, Y is the matrix of observed responses, X and Z are the design matrices for fixed effects and mixed effects respectively, $\beta$ is the matrix of unknown fixed parameters, $b$ is the matrix of unknown random parameters, and $\epsilon$ is the vector of unobservable model errors. Because we apply weights to observations based on age, the least squares estimates for the fixed effects model parameters in this model are obtained by the following matrix formula, when we subtract away the random effects:

3 $$\widehat{{\beta }}=\left[\begin{array}{c}{\beta }_{0}\\ \vdots \\ {\beta }_{v}\end{array}\right]={(X^{\prime}\bullet W\bullet X)}^{-1}X^{\prime}\bullet W\bullet Y$$

where *Y* is the matrix of observed responses, ${\beta }_{0}$ to ${\beta }_{v}$ are the regression estimates for the intercept and all fixed effects, *X* is the design matrix, and *W* is the diagonal matrix of weights. The weights we apply to observations in the model are calculated in three steps. In step 1 the vector of ages is weighted as a function of center (age of interest) and shoulder (reflecting kurtosis in the distribution of weights):

4 $${w}_{1}=1-{\left|c-x\right|}^{s}$$

In this notation, ${w}_{1}$ is the weights vector after step 1, $x$ is the vector of ages, *c* is the center of the weights, and *s* is the shoulder. In the second step, the weights vector from step 1 is scaled, by applying a min–max scalar:

5 $${w}_{2}=({w}_{1}-min({w}_{1}))/(max({w}_{1})-min({w}_{1}))$$

**Fig. 5:**
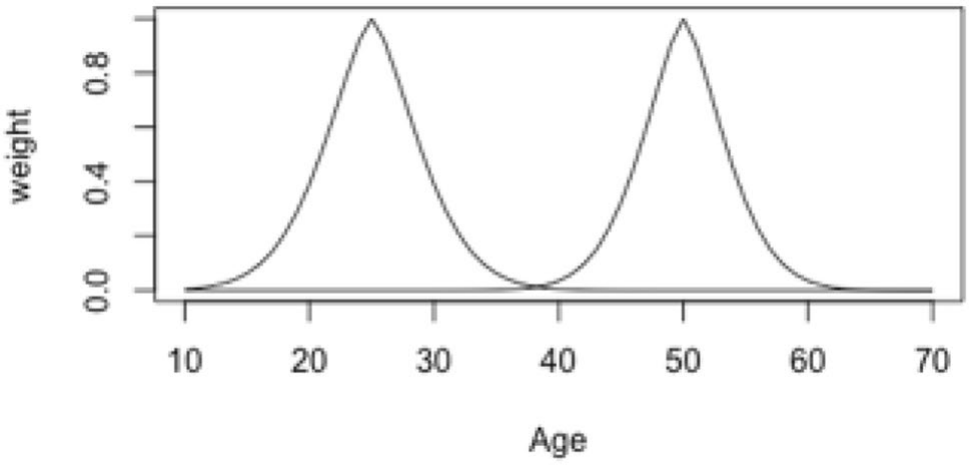
Example of weights with centers at ages 25 and 50

In this notation, ${w}_{2}$ is the weights vector after step 2, ${w}_{1}$ is the weights vector after step 1. Third, the desired decay is applied to the scaled vector *w*_*2*_, and the final diagonal matrix of weights is calculated:

6 $$W=diag({{w}_{2}}^{{e}^{k}})$$

where *W* is the final diagonal matrix of weights, *w*_*2*_ is the weights vector after step 2 and *e*^k^ represents the decay in the distribution of weights. In this approach, we opted for a shoulder of 1.5, and a decay of 25. See Fig. 5 for an example of the weights for ages 25 and 50. In general, a wider distribution of weights smooths the sample size and age-specific effects more, while a narrower distribution is more sensitive to fluctuations in sample size and age effects. We ran supplemental analyses to test the impact of wider and narrower age weight distributions (see Supplements Fig. 2 and 3).

All continuous fixed effects and the IRT aggression scores were standardized before the analyses. We employed bootstrapping to assess the robustness of the model estimated standard error. The approach was to sample complete families with replacement from the original data, 100 samples for each age-analysis. We found that model implied standard errors were slightly underestimated. Therefore, 95% confidence intervals reported in the results were calculated with the bootstrap standard errors. Significance is implied when the empirical (bootstrap) 95% confidence intervals do not intersect zero.

## Results

**Fig. 6:**
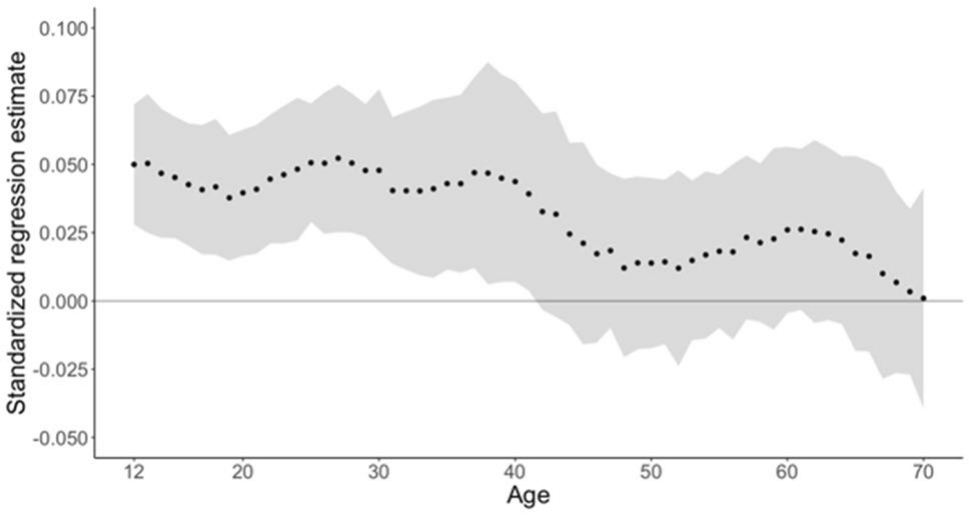
Dutch data: standardized regression estimates for the effect of the PGS with bootstrapped 95% confidence intervals (as grey banners)

In the total genotyped Dutch sample (N_observations_ = 29,454, N_individuals_ = 13,471), we first analyzed all data together without weighting the data. In this approach, the PGS was significantly associated with aggression, *β* = 0.05, *SE* = 0.01, *pseudo R*^*2*^ = 0.002, P < 0.001. Next, we ran the age-specific age models with rolling weights. These analyses showed that the PGS was significantly related to IRT aggression from age 12 to age 41 (*β* = 0.04–0.05; Fig. 6, Supplements Table I). After age 41, the effect of the PGS decreases, with confidence intervals that are close to, or below zero. The highest estimates for the effect of the PGSs are at around age 28. Supplemental analyses with wider and narrower age weights show that the width of the distribution affects the smoothing of the results, meaning that a narrower distribution of weights leads to more defined differences in PGS effects between ages (see Supplements Figs. 1 and 2).

**Fig. 7:**
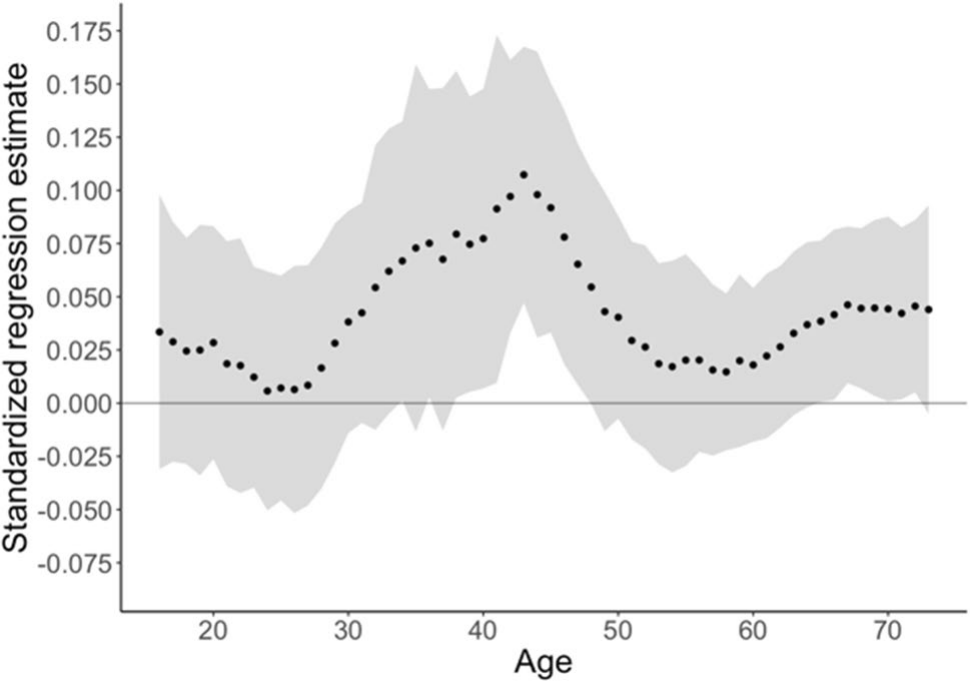
Australian data: standardized regression estimates for the effect of the PGS with bootstrapped 95% confidence intervals (as grey banners)

In the total genotyped Australian sample without rolling weights (N_observations_ = 7231, N_individuals_ = 5, 28), the PGS was significantly associated with adult and adolescent aggression, *β* = 0.04, *SE* = 0.01, *pseudo R*^2^ = 0.002, P = 0.002. The specific age analyses from the model with rolling weights suggested a different association pattern across age (see Fig. 7, Supplements Table II) compared to the Dutch sample. The results did not indicate a downward trend in the regression estimates. Instead, the PGS was significantly related to aggression at ages 38 to 48 (*β* = 0.04–0.11).

In the Australian cohort we also investigated whether there are differences in prediction for the four subscales of the Buss Perry Aggression questionnaire (i.e. Physical aggression, Verbal aggression, Anger, and Hostility). The trends are very similar across all subscales. The peak around age 45 is clearly present for all subscales, albeit to a slightly lesser extent in verbal aggression. For figures of the regression estimates from the Buss Perry subscales see Supplements Fig. 3.

## Discussion

In this study we introduce a new method to investigate the effect of a PGS across levels of a continuous moderating variable, in this case age. The approach is to run a linear model for each age in years present in the data. In each analysis, the phenotype information is weighted, with weights that are centered at the focus age and decay further away from that center. The strength of this method is that with each age analysis, information on proximal ages is taken into account, mitigating the risk that sample size differences at different ages will drive the effects we find. We applied this approach to assess the association between childhood aggression PGSs and aggression across the life-course, in two cohorts from The Netherlands and Australia.

In The Netherlands, the PGS was significantly related to aggression at ages 12 to 41 years. The effect of the PGS decreased from age 41–70 years. In the smaller Australian cohort, the effect of the PGS was significant at ages 38 to 48. Sample sizes at the peak of the PGS effect in Australia were between *N* = 193 and *N* = 868 (calculated as sum of weights, see Supplements Table II), which was relatively small. Because of these sample sizes, we should interpret this peak in effects with caution; for example, it could be driven by a small number of individuals that are not representative of the population. Effect sizes are small in both cohorts, with just under 0.2% explained variance in the full non-weighted models. We expect that effect sizes will increase as discovery GWAS sample sizes increase.

Although the effects are small, these results are the first indication from a molecular genetics perspective that genetic influences drive part of the continuity and stability of aggressive behavior, and that genetic effects in childhood persist across life, and thus across situations. This suggests that throughout people’s lives, most notably in the Dutch context, developmental changes in individuals only slightly impact the polygenic effects on aggression that were apparent in childhood. These results correspond with findings from longitudinal twin studies on the stability of aggression in children (van Beijsterveldt et al. 2003; Eley et al. 2003; Porsch et al. 2016), and a twin study on the stability of externalizing psychopathology in adults (Gustavson et al. 2020), where genetic factors accounted for a large part of the stability over time. Van Beijsterveldt et al. (2003) also demonstrated that new genetic influences can contribute to stability of aggression across different ages. As such, the continuity of polygenic effects across the life-course covers only part of the genetic influences on the stability of aggression across the life-course.

A strength of this study is that we investigate the association between the PGS and aggression in two cohorts. This also implies, if we want to compare our results across cohorts, that there are several considerations, including differences in phenotyping, the impact of the discovery data, cultural differences between societies, and genetic differences between populations that may limit the feasibility of a reliable comparison. In the Dutch cohort, phenotyping was done with the ASEBA self-report questionnaires. In the Australian cohort, phenotyping was done with the Buss Perry Aggression self-report questionnaire. Differences in phenotyping are somewhat mitigated by using an IRT latent variable as dependent variable, instead of a sum score. Because different measurement instruments are used, this somewhat limits how we draw conclusions from the differences we found between the cohorts. A large percentage of participants in the discovery childhood aggression GWAMA from which we calculated our PGSs, were also scored with the ASEBA instruments, i.e. self-, parent- and teacher-reports (Ip et al. 2021), which relate directly to the ASEBA self-report questionnaires used in the current Dutch sample. Correlations between self-, parent- and teacher-reports were not very high in Ip and colleagues’ GWAMA. Still, we expect slightly greater power in the Dutch cohort compared to the Australian cohort, based on the similarities in the measurement instrument. Another potential source of dissimilarity is that the cohorts were not phenotyped in the same years. In The Netherlands, participants were phenotyped between 1991 and 2014, in Australia between 2014 and 2020, i.e. individuals that were phenotyped at the same age, are often not phenotyped in the same year. Van der Laan et al. (in press) show that self-reported aggression in The Netherlands declined from 1991 to 2015. Thus, differences between cohorts may be influenced by time effects. Another difference in phenotyping between the cohorts was that missing data (< 20%) were imputed prior to calculating IRT scores, while in the Dutch sample missing data (< 20%) were handled by the IRT models when calculating the scores.

The two discovery GWAMAs for the Dutch and Australian cohorts were also not identical. For The Netherlands, discovery data excluded all NTR participants. For Australia, discovery data excluded all Brisbane Longitudinal Twin Study and Prospective Imaging Study of Ageing participants. We opted for this strategy because leaving participants from both cohorts out of the discovery data would mean an unnecessary decrease in sample size. The differences in the discovery GWAMAs means that the PGSs in both samples are calculated based on overlapping, but not identical information. This may have resulted in slight differences in association between cohorts, although respectively only 5 and 17% of the Dutch and Australian discovery samples was not shared.

More generally, even if phenotyping is similar, cultural and genetic differences between populations can affect the magnitude, and thereby the merit, of PGS predictions. Cultural norms may influence aggression directly, and thereby moderate the effect of PGSs on aggression. One way to measure the effects of cultural differences on traits is by assessing generalizability of the measurement instruments via Confirmatory Factor Analysis (CFA), using the framework of measurement invariance (Millsap 2012). By assessing measurement invariance, we can test whether we measure the same underlying psychopathological trait when studying different societies. Because the Australian and Dutch cohorts did not phenotype by the same instruments, we cannot test measurement invariance directly in our sample. However, measurement invariance for the ASEBA self-report questionnaires is well documented. CFA of the eight-syndrome structure of the youth self-report, originally derived from a U.S. general population sample, plus clinically referred youths from Australia, England, and the United States (Achenbach and Rescorla 2001), fits YSR data from a wide range of societies (Ivanova et al. 2007). Fit indices were almost identical between Australia, RMSEA = 0.042 and The Netherlands, RMSEA = 0.040. Ivanova and colleagues (2015) also investigated generalizability of the eight-syndrome structure of the adult self-report (ASR) in 29 societies. Although The Netherlands and Australia were not included, model fit was good for all samples, with fit indices very similar to those found in the YSR CFAs. Generalizability of the Buss Perry aggression questionnaire has been less extensively studied, but validation is well documented for young Western adults (see for an overview Gerevich et al. 2007). Generalizability has been questioned for older and more diverse samples, but the four-factor structure (Physical aggression, Verbal Aggression, Hostility, and Anger) did replicate in a sample of Chilean students (Valdivia-Peralta et al. 2014), a slimmed down, translated 12 item version (Bryant and Smith 2001) replicated well in a sample of Hong Kong Chinese (Maxwell 2007), and all factors except Anger replicated in Hungarian adults with a mean age of 46.6 years (Gerevich et al. 2007).

Another source of differences between the population cohorts may be genetic heterogeneity. Based on genetic marker data, there are several ways to assess the comparability of samples from different populations. In an early comparison based on DNA tandem repeat polymorphisms from The Netherlands and Australia, Sullivan et al. (2006) estimated the fixation index (F_ST_)—the percentage genetic variability attributable to genetic differences between cohorts—to be only 0.3%. The empirical variability between the Australian and Dutch cohorts was in fact smaller than for a combination of European samples (F_ST_ = 0.8%; Rosenberg 2021). More recently, Beck and colleagues (2019) studied interpopulation stratification between cohorts from The Netherlands and Australia, by analyzing genome wide SNP data. Negligible interpopulation stratification was confirmed by visualizing uncorrelated principal components and F_ST_ estimations of 0.05%. These findings should not be surprising, given the colonization history of Australia via immigrants from the UK, who are genetically very similar to Dutch individuals, as previously described with similarities in Y-chromosome haplogroups (Rosser et al. 2000).

At the moment, this genetic similarity between cohorts included in genomic studies is the rule rather than the exception. Around 78% of individuals included in GWAS are from European ancestry (EA; Buniello et al. 2019). The lack of individuals from non-EA populations included in GWAS, means that it is often unclear to what extent genetic effects generalize to diverse populations. Carlson and colleagues (2013) demonstrated that in non-EA populations, most GWAS-identified variants have allelic associations in the same direction as in EA populations, with none showing a statistically significant effect in the opposite direction. However, 25% of tagSNPs had significantly different effect sizes in at least one non-EA population, most frequent in African Americans, with all differential effects diluted towards zero. Thus, associations between the PGS based on the Ip et al. (2021) discovery and aggression might be weaker in non-EA populations, as has been seen for other traits, such as obesity/BMI (Domingue et al. 2014; Belsky et al. 2013; Ware et al. 2017), height (Ware et al. 2017), educational attainment (Domingue et al. 2015; Ware et al. 2017; Lee et al. 2018), schizophrenia (Vilhalmsson et al. 2015; Ware et al. 2017; Vassos et al. 2017), and breast cancer (Ho et al. 2020). This means that advances through GWAS and PGS studies tended to benefit EA populations more than non-EA populations, especially when predicting health outcomes (Martin et al. 2019). The overrepresentation of EA participants in GWAS is partly because admixed populations were long considered inconvenient in gene discovery studies, as this led to population stratification issues. However, due to advances in GWAS methods, populations with mixed genetic backgrounds can now be included in GWAS to obtain accurate estimates of SNP effects, boost power, and improve fine-mapping of effects by leveraging linkage disequilibrium differences (Asimit et al. 2016; Atkinson et al. 2020). Beside the general benefits to gene discovery studies, the inclusion of diverse genetic backgrounds will improve our understanding of genetic liability across diverse populations, as demonstrated for example in a study of glycemic traits (Chen et al. 2021), where 30% of the participants were of non-European ancestry.

In summary, we investigated the continuity of polygenic effects on aggression across the life-course, in two cohorts from The Netherlands and Australia, with a novel weighted mixed effects regression approach. Our results suggest that the same genetic factors that explain part of the individual differences in aggression in childhood, also explain individual differences in adolescents and adults. The new method we employed shows promise in modeling genetic effects across levels of a continuous moderating variable, in way that smooths any possible effects due to sample size differences between those levels. The possibilities of reliably comparing results between the Dutch and Australian cohorts were limited because of differences in phenotyping and GWAMA discovery samples. When studying genetic liability in different populations, there are two main considerations: cultural/environmental differences and genetic differences. If we are interested in studying differences in genetic effects between populations with different cultural norms and environments, the optimal design is to look at populations with similar genetic backgrounds. For this to work, phenotyping has to be standardized across populations. To better understand genetic effects in diverse genetic populations, we need gene discovery studies that include diverse populations, so that predictions in non-EA populations are not dependent on EA discovery samples.

## Supplementary Information

Below is the link to the electronic supplementary material.

Supplementary file1 (DOCX 1664 kb)

## Data Availability

Available upon request.

## Code Availability

Please contact first author.

## References

[CR1] Abdellaoui A, Hottenga JJ, De Knijff P, Nivard MG, Xiao X, Scheet P (2013). Population structure, migration, and diversifying selection in The Netherlands. Eur J Hum Genet.

[CR2] Achenbach TM, Rescorla LA (2001). Manual for the ASEBA school-age forms & profiles.

[CR3] Achenbach TM, Rescorla LA (2003). Manual for the ASEBA adult forms & profiles.

[CR4] Achenbach TM, Ivanova MY, Rescorla LA (2017). Empirically based assessment and taxonomy of psychopathology for ages 11⁄2-90+ years: developmental, multi-informant, and multicultural findings. Compr Psychiatry.

[CR5] Alink LR, Mesman J, Van Zeijl J, Stolk M, Juffer F, Koot H (2006). The early childhood aggression curve: development of physical aggression in 10- to 50-month-old children. Child Dev.

[CR6] Anderson CA, Bushman BJ (2002). Human aggression. Annu Rev Psychol.

[CR7] Asimit JL, Hatzikotoulas K, McCarthy M, Morris AP, Zeggini E (2016). Trans-ethnic study design approaches for fine-mapping. Eur J Hum Genet.

[CR8] Atkinson EG, Maihofer AX, Kanai M, Martin AR, Karczewski KJ, Santoro ML, et al. (2020) Tractor: a framework allowing for improved inclusion of admixed individuals in large-scale association studies. https://www.biorxiv.org/content/10.1101/2020.05.17.100727v1

[CR9] Baron RA, Richardson DR (1994). Human aggression.

[CR10] Bates D, Maechler M, Bolker B, Walker S (2015). Fitting linear mixed-effects models using lme4. J Stat Softw.

[CR11] Beck J, Hottenga JJ, Mbarek H, Finnicum C, Ehli E, Hur Y (2019). Genetic similarity assessment of twin-family populations by custom-designed genotyping array. Twin Res Hum Genet.

[CR12] Belsky DW, Moffitt TE, Sugden K, Williams B, Houts R, McCarthy J, Caspi A (2013). Development and evaluation of a genetic risk score for obesity. Biodemography Soc Biol.

[CR13] Boomsma DI (2015). Aggression in children: unravelling the interplay of genes and environment through (epi)genetics and metabolomics. J Ped Neo Ind Med.

[CR14] Boomsma DI, Vink JM, van Beijsterveldt CEM, de Geus EJC, Beem AL, Mulder EJCM (2002). Netherlands twin register: a focus on longitudinal research. Twin Res.

[CR15] Boomsma DI, de Geus EJC, Vink JM, Stubbe JH, Distel MA, Hottenga JJ, Willemsen G (2006). Netherlands Twin Register: from twins to twin families. Twin Res Hum Genet.

[CR16] Buniello A, MacArthur J, Cerezo M, Harris LW, Hayhurst J, Malangone C (2019). The NHGRI-EBI GWAS Catalog of published genome-wide association studies, targeted arrays and summary statistics 2019. Nucleic Acids Res.

[CR17] Buss AH, Perry M (1992). The aggression questionnaire. J Pers Soc Psychol.

[CR18] Cairns RB, Cairns BD (1994). Life lines and risks: pathways of youth in our time.

[CR19] Cairns RB, Cairns BD, Neckerman H, Ferguson L, Gariépy J (1989). Growth and aggression: I. Childhood to early adolescence. Dev Psychol.

[CR20] Carlson CS, Matise TC, North KE, Haiman CA, Fesinmeyer MD, Buyske S (2013). Generalization and dilution of association results from european gwas in populations of non-european ancestry: the page study. PLos Biol.

[CR21] Chalmers RP (2012). mirt: a multidimensional item response theory package for the R environment. J Stat Softw.

[CR22] Chen J, Spracklen CN, Marenne G, Varshney A, Corbin LJ, Luan J et al (2021). The trans-ancestral genomic architecture of glycemic traits. Nat Genet.

[CR23] Das S, Forer L, Schönherr S, Sidore C, Locke AE, Vrieze SI (2016). Next-generation genotype imputation service and methods. Nat Genet.

[CR24] Domingue BW, Belsky DW, Harris KM, Smolen A, McQueen MB, Boardman JD (2014). Polygenic risk predicts obesity in both white and black young adults. PLoS ONE.

[CR25] Domingue BW, Belsky D, Conley D, Harris KM, Boardman JD (2015). Polygenic influence on educational attainment: new evidence from The National Longitudinal Study of Adolescent to Adult Health. AERA Open.

[CR26] Eley TC, Lichtenstein P, Moffitt TE (2003). A longitudinal behavioral genetic analysis of the etiology of aggressive and nonaggressive antisocial behavior. Dev Psychopathol.

[CR27] Embretson SE, Reise SP (2000). Item response theory for psychologists.

[CR29] Farrington DP (1989). Early predictors of adolescent aggression and adult violence. Violence Vict.

[CR30] Gerevich J, Bácskai E, Czobor P (2007). The generalizability of the buss-perry aggression questionnaire. Int J Methods Psychiatr Res.

[CR31] Gustavson DE, Franz CE, Panizzon MS, Lyons MJ, Kremen WS (2020). Internalizing and externalizing psychopathology in middle age: genetic and environmental architecture and stability of symptoms over 15 to 20 years. Psychol Med.

[CR32] Ho W-K, Tan M-M, Mavaddat N, Tai M-C, Mariapun S, Li J (2020). European *polygenic risk score for prediction of breast cancer shows similar performance in asian women. Nat Commun.

[CR33] Huesmann LR, Dubow EF, Boxer P (2009). Continuity of aggression from childhood to early adulthood as a predictor of life outcomes: implications for the adolescent-limited and life-course-persistent models. Aggr Behav.

[CR34] Ip HF, van der Laan CM, Brikell I, Sánchez-Mora C, Nolte IM, Pourcain B (2021). Genetic association study of childhood aggression across raters, instruments, and age. Transl Psychiatry.

[CR35] Ivanova MY, Achenbach TM, Rescorla LA, Dumenci L, Almqvist F, Bilenberg N (2007). The generalizability of the youth self-report syndrome structure in 23 societies. J Consult Clin Psychol.

[CR36] Ivanova MY, Achenbach TM, Rescorla LA, Turner LV, Ahmeti-Pronaj A, Au A (2015). Syndromes of self-reported psychopathology for ages 18–59 in 29 societies. J Psychopathol Behav Assess.

[CR37] Karriker-Jaffe K, Foshee V, Ennett S, Suchindran C (2008). The development of aggression during adolescence: sex differences in trajectories of physical and social aggression among youth in rural areas. J Abnorm Child Psychol.

[CR38] Labella M, Masten A (2018). Family influences on the development of aggression and violence. Curr Opin Psychol.

[CR39] Lee JJ, Wedow R, Okbay A, Kong E, Maghzian O, Zacher M (2018). Gene discovery and polygenic prediction from a genome-wide association study of educational attainment in 1.1 million individuals. Nat Genet.

[CR40] Ligthart L, van Beijsterveldt CEM, Kevenaar ST, de Zeeuw E, van Bergen E, Bruins S (2019). The Netherlands Twin Register: longitudinal research based on twin research and twin-family designs. Twin Res Hum Genet.

[CR41] Lloyd-Jones LR, Zeng J, Sidorenko J, Yengo L, Moser G, Kemper KE (2019). Improved polygenic prediction by Bayesian multiple regression on summary statistics. Nat Commun.

[CR42] Loeber R, Stouthamer-Loeber M (1998). Development of juvenile aggression and violence. Some common misconceptions and controversies. Am Psychol.

[CR43] Lupton MK, Robinson GA, Adam RJ, Rose S, Byrne GJ, Salvado O (2021). A prospective cohort study of prodromal Alzheimer′s disease: prospective imaging study of ageing: genes, brain and behaviour (PISA). NeuroImage.

[CR44] Manichaikul A, Mychaleckyj JC, Rich SS, Daly K, Sale M, Chen WM (2010). Robust relationship inference in genome-wide association studies. Bioinformatics (Oxford, England).

[CR45] Martin AR, Kanai M, Kamatani Y, Okada Y, Neale BM, Daly MJ (2019). Clinical use of current polygenic risk scores may exacerbate health disparities. Nat Genet.

[CR46] Maxwell JP (2007). Development and preliminary validation of a chinese version of the buss-perry aggression questionnaire in a population of hong kong chinese. J Pers Assess.

[CR47] Millsap R (2012). Statistical approaches to measurement invariance.

[CR48] Mitchell BL, Campos AI, Rentería ME, Parker R (2019). Twenty-five and up (25Up) study: a new wave of the Brisbane Longitudinal Twin Study. Twin Res Hum Genet.

[CR49] Mitchell BL, Kirk KM, McAloney K, Wright MJ, Davenport TA (2020). 16up: outline of a study investigating wellbeing and information and communication technology use in adolescent twins. Twin Res Hum Genet.

[CR50] Moffitt TE (1993). Adolescence-limited and life-course-persistent antisocial behavior: a developmental taxonomy. Psychol Rev.

[CR51] Morosoli JJ (2020) Overcoming genetic determinism: A psychological perspective on public understanding of human complex trait genetics. (Unpublished doctoral dissertation). University of Queensland, Brisbane, Australia.

[CR52] Murray J, Farrington DP (2010). Risk factors for conduct disorder and delinquency: key findings from longitudinal studies. Can J Psychiatry.

[CR53] Odintsova VV, Roetman PJ, Ip HF, Pool R, Van der Laan CM, Tona K-D (2019). Genomics of human aggression: current state of genome-wide studies and an automated systematic review tool. Psychiatr Genet.

[CR54] Porsch RM, Middeldorp CM, Cherny SS, Krapohl E, van Beijsterveldt CEM, Loukola A (2016). Longitudinal heritability of childhood aggression. Am J Med Genet B Neuropsychiatr Genet.

[CR55] Pulkkinen L, Pitkänen T (1993). Contiuities in aggressive behavior from childhood to adulthood. Aggr Behav.

[CR56] Purcell S, Neale B, Todd-Brown K, Thomas L, Ferreira MAR, Bender D (2007). PLINK: a toolset for whole-genome association and population-based linkage analysis. Am J Hum Genet.

[CR57] R Core Team (2017). R: a language and environment for statistical computing.

[CR75] Rosenberg NA (2021). A population-genetic perspective on the similarities and differences among worldwide human populations. Hum Biol.

[CR58] Rosenberg NA, Pritchard JK, Weber JL, Cann HM, Kidd KK, Zhivotovsky LA, Feldman MW (2002). Genetic structure of human populations. Science.

[CR59] Rosser ZH, Zerjal T, Hurles ME, Adojaan M, Alavantic D, Amorim A (2000). Y-chromosomal diversity in Europe is clinal and influenced primarily by geography, rather than by language. Am J Hum Genet.

[CR60] Tolan PH, Dodge K, Rutter M, Tolan PH, Leventhal BL (2013). Tracking the multiple pathways of parent and family influence on disruptive behavior disorders. Disruptive behavior disorders.

[CR61] Tremblay RE (2010). Developmental origins of disruptive behaviour problems: the ‘original sin’ hypothesis, epigenetics and their consequences for prevention. J Child Psychol Psychiatry.

[CR62] Tremblay RE, Nagin DS, Séguin JR, Zoccolillo M, Zelazo PD, Boivin M (2004). Physical aggression during early childhood: trajectories and predictors. Pediatrics.

[CR63] Tuvblad C, Baker LA (2011). Human aggression across the lifespan: genetic propensities and environmental moderators. Adv Genet.

[CR64] Underwood MK (2003). Social aggression among girls.

[CR65] Valdivia-Peralta M, Fonseca-Pedrero E, González-Bravo L, Lemos-Giráldez S (2014). Psychometric properties of the AQ aggression scale in Chilean students. Psicothema.

[CR67] van Beijsterveldt CEM, Bartels M, Hudziak JJ, Boomsma DI (2003). Causes of stability of aggression from early childhood to adolescence: a longitudinal genetic analysis in Dutch twins. Behav Genet.

[CR66] van Buuren S, Groothuis-Oudshoorn K (2011). Mice: multivariate imputation by chained equations in R. J Stat Softw.

[CR28] van der Laan CM, van de Weijer SGA, Nivard MG, Boomsma DI (in press) Familial clustering of trends in aggression. J Quant Criminol

[CR68] Vassos E, Di Forti M, Coleman J, Iyegbe C, Prata D, Euesden J (2017). An examination of polygenic score risk prediction in individuals with first-episode psychosis. Biol Psychiat.

[CR69] Veroude K, Zhang-James Y, Fernàndez-Castillo N, Bakker MJ, Cormand B, Faraone SV (2016). Genetics of aggressive behavior: an overview. Am J Med Genet Part B Neuropsychiatr Genet.

[CR70] Vilhjálmsson BJ, Yang J, Finucane HK, Gusev A, Lindström S, Ripke S (2015). Modeling linkage disequilibrium increases accuracy of polygenic risk scores. Am J Hum Genet.

[CR71] Vuoksimaa E, Rose RJ, Pulkkinen L, Palviainen T, Rimfeld K, Lundström S (2021). Higher aggression is related to poorer academic performance in compulsory education. J Child Psychol Psychiatry.

[CR76] Waltes R, Chiocchetti AG, Freitag CM (2016). The neurobiological basis of human aggression: a review on genetic and epigenetic mechanisms. Am J Med Genet B Neuropsychiatr Genet.

[CR72] Ware, EB, Schmitz, LL, Faul, JD, Gard, A, Mitchell, C, Smith, JA, et al (2017) Heterogeneity in polygenic scores for common human traits (bioRxiv). 10.1101/106062

[CR73] Wray NR, Lee SH, Mehta D, Vinkhuyzen AAE, Dudbridge F, Middeldorp CM (2014). Research review: polygenic methods and their application to psychiatric traits. J Child Psychol Psychiatry.

[CR74] Wright MJ, Martin NG (2004). Brisbane Adolescent Twin Study: outline of study methods and research projects. Aust J Psychol.

