# Supplementary material for: Continuity of Genetic Risk for Aggressive Behavior Across the Life-Course
Source: Behav Genet. 2021 Aug 14;51(5):592–606. doi: 10.1007/s10519-021-10076-6 (PMC8390412; doi:10.1007/s10519-021-10076-6)
Supplement: Supplementary file 1 — Supplementary file1 (DOCX 1664 kb) [file 10519_2021_10076_MOESM1_ESM.docx]

**Continuity of Genetic Risk for Aggressive Behavior Across the Life-Course**

**Supplements**

1. Wide weights distribution B. Narrow weights distribution


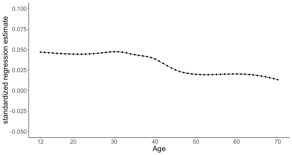

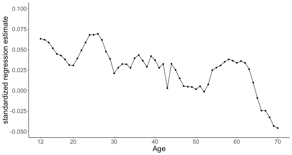


Figure 1.  *The Netherlands. Regression estimates for PGS p< 0.1 with two different weight distributions for each age. On the left: results from models with a wider distribution of weights, with low sensitivity to specific age effects. On the right: results from models with a narrower distribution of weights, with high sensitivity to specific age effects. See Figure 3 for an example of the differences in weight distributions.*


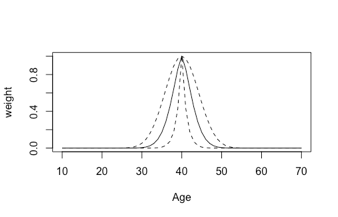


Figure 2. *Example* *of the wider and narrower distributions at age 40, used for Supplements Figure 1. The solid line reflects the distribution of weights used in our main analyses. The dotted lines represent the wider and narrower distributions.*

A. Physical Aggression B. Hostility


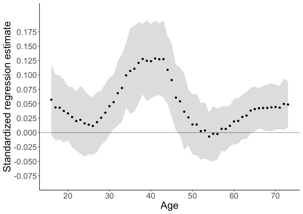

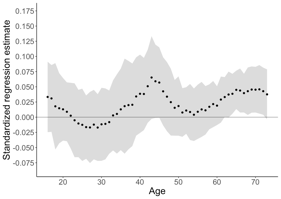


C. Anger D. Verbal Aggression


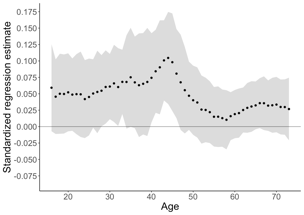

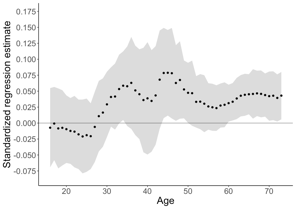


Figure 3. *Australia: standardized regression estimates with 95% confidence intervals (as grey banners) from age weighted mixed effects models with Buss Perry subscales as outcome variables (Physical Aggression, Hostility, Anger, Verbal Aggression)*.

| Table I. The Netherlands. Results from age-weighted models. | | | | | |
| --- | --- | --- | --- | --- | --- |
|  |  |  | Bootstrapped 95% CI | |  |
| AGE | *β* | Bootstrapped *β* | Lower | Upper | *N* |
| 12 | 0.05 | 0.05 | 0.03 | 0.07 | 6032 |
| 13 | 0.05 | 0.05 | 0.02 | 0.08 | 7142 |
| 14 | 0.05 | 0.05 | 0.02 | 0.07 | 8324 |
| 15 | 0.05 | 0.05 | 0.02 | 0.07 | 9478 |
| 16 | 0.04 | 0.04 | 0.02 | 0.07 | 10428 |
| 17 | 0.04 | 0.04 | 0.02 | 0.06 | 11147 |
| 18 | 0.04 | 0.04 | 0.02 | 0.07 | 11506 |
| 19 | 0.04 | 0.04 | 0.01 | 0.06 | 11531 |
| 20 | 0.04 | 0.04 | 0.02 | 0.06 | 11236 |
| 21 | 0.04 | 0.04 | 0.02 | 0.06 | 10706 |
| 22 | 0.05 | 0.04 | 0.02 | 0.07 | 9997 |
| 23 | 0.05 | 0.05 | 0.02 | 0.07 | 9176 |
| 24 | 0.05 | 0.05 | 0.02 | 0.07 | 8302 |
| 25 | 0.05 | 0.05 | 0.03 | 0.07 | 7415 |
| 26 | 0.05 | 0.05 | 0.02 | 0.08 | 6559 |
| 27 | 0.05 | 0.05 | 0.03 | 0.08 | 5770 |
| 28 | 0.05 | 0.05 | 0.03 | 0.08 | 5058 |
| 29 | 0.05 | 0.05 | 0.02 | 0.07 | 4461 |
| 30 | 0.05 | 0.05 | 0.02 | 0.08 | 3980 |
| 31 | 0.04 | 0.04 | 0.01 | 0.07 | 3604 |
| 32 | 0.04 | 0.04 | 0.01 | 0.07 | 3332 |
| 33 | 0.04 | 0.04 | 0.01 | 0.07 | 3146 |
| 34 | 0.04 | 0.04 | 0.01 | 0.07 | 3005 |
| 35 | 0.04 | 0.04 | 0.01 | 0.07 | 2895 |
| 36 | 0.04 | 0.04 | 0.01 | 0.08 | 2801 |
| 37 | 0.04 | 0.05 | 0.01 | 0.08 | 2697 |
| 38 | 0.04 | 0.05 | 0.01 | 0.09 | 2570 |
| 39 | 0.04 | 0.04 | 0.01 | 0.08 | 2426 |
| 40 | 0.04 | 0.04 | 0.01 | 0.08 | 2269 |
| 41 | 0.03 | 0.04 | 0 | 0.07 | 2106 |
| 42 | 0.03 | 0.03 | 0 | 0.07 | 2083 |
| 43 | 0.03 | 0.03 | -0.01 | 0.07 | 2066 |
| 44 | 0.02 | 0.02 | -0.01 | 0.06 | 2061 |
| 45 | 0.02 | 0.02 | -0.02 | 0.06 | 2081 |
| 46 | 0.02 | 0.02 | -0.02 | 0.05 | 2129 |
| 47 | 0.02 | 0.02 | -0.01 | 0.05 | 2207 |
| 48 | 0.01 | 0.01 | -0.02 | 0.04 | 2309 |
| 49 | 0.01 | 0.01 | -0.02 | 0.05 | 2428 |
| 50 | 0.01 | 0.01 | -0.02 | 0.05 | 2553 |
| 51 | 0.01 | 0.01 | -0.02 | 0.04 | 2674 |
| 52 | 0.01 | 0.01 | -0.02 | 0.05 | 2783 |
| 53 | 0.02 | 0.01 | -0.01 | 0.04 | 2875 |
| 54 | 0.02 | 0.02 | -0.01 | 0.05 | 2943 |
| 55 | 0.02 | 0.02 | -0.01 | 0.05 | 2987 |
| 56 | 0.02 | 0.02 | -0.01 | 0.05 | 3006 |
| 57 | 0.02 | 0.02 | -0.01 | 0.05 | 3001 |
| 58 | 0.02 | 0.02 | -0.01 | 0.05 | 2968 |
| 59 | 0.03 | 0.02 | -0.01 | 0.06 | 2905 |
| 60 | 0.03 | 0.03 | 0 | 0.06 | 2807 |
| 61 | 0.03 | 0.03 | 0 | 0.06 | 2676 |
| 62 | 0.03 | 0.03 | -0.01 | 0.06 | 2513 |
| 63 | 0.02 | 0.02 | -0.01 | 0.06 | 2317 |
| 64 | 0.02 | 0.02 | -0.01 | 0.05 | 2101 |
| 65 | 0.02 | 0.02 | -0.02 | 0.05 | 1876 |
| 66 | 0.01 | 0.02 | -0.02 | 0.05 | 1648 |
| 67 | 0.01 | 0.01 | -0.03 | 0.05 | 1429 |
| 68 | 0 | 0.01 | -0.03 | 0.04 | 1224 |
| 69 | 0 | 0 | -0.03 | 0.03 | 1036 |
| 70 | 0 | 0 | -0.04 | 0.04 | 866 |
| Note: *β*= standardized regression estimate, Bootstrapped *β*= Bootstrapped standardized regression estimate, Bootstrapped 95% CI= empirical 95% confidence intervals, *N*= sum of regression weights. | | | | | |

| Table II. Australia. Results from age-weighted models. | | | | | |
| --- | --- | --- | --- | --- | --- |
|  |  |  | Bootstrapped 95% CI | |  |
| AGE | *β* | Bootstrapped *β* | Lower | Upper | *N* |
| 16 | 0.03 | 0.03 | -0.03 | 0.1 | 754 |
| 17 | 0.03 | 0.03 | -0.03 | 0.09 | 829 |
| 18 | 0.03 | 0.02 | -0.03 | 0.08 | 898 |
| 19 | 0.03 | 0.02 | -0.03 | 0.08 | 968 |
| 20 | 0.03 | 0.03 | -0.03 | 0.08 | 1047 |
| 21 | 0.02 | 0.02 | -0.04 | 0.08 | 1140 |
| 22 | 0.02 | 0.02 | -0.04 | 0.08 | 1249 |
| 23 | 0.02 | 0.01 | -0.04 | 0.06 | 1373 |
| 24 | 0.01 | 0.01 | -0.05 | 0.06 | 1498 |
| 25 | 0.01 | 0.01 | -0.05 | 0.06 | 1611 |
| 26 | 0.01 | 0.01 | -0.05 | 0.06 | 1704 |
| 27 | 0.01 | 0.01 | -0.05 | 0.06 | 1770 |
| 28 | 0.02 | 0.02 | -0.04 | 0.07 | 1806 |
| 29 | 0.03 | 0.03 | -0.03 | 0.08 | 1808 |
| 30 | 0.03 | 0.04 | -0.01 | 0.09 | 1785 |
| 31 | 0.04 | 0.04 | -0.01 | 0.09 | 1744 |
| 32 | 0.05 | 0.05 | -0.01 | 0.12 | 1688 |
| 33 | 0.06 | 0.06 | 0 | 0.13 | 1620 |
| 34 | 0.06 | 0.07 | 0 | 0.13 | 1526 |
| 35 | 0.07 | 0.07 | -0.01 | 0.16 | 1405 |
| 36 | 0.07 | 0.08 | 0 | 0.15 | 1250 |
| 37 | 0.08 | 0.07 | -0.01 | 0.15 | 1067 |
| 38 | 0.08 | 0.08 | 0 | 0.16 | 868 |
| 39 | 0.08 | 0.07 | 0.01 | 0.14 | 672 |
| 40 | 0.09 | 0.08 | 0.01 | 0.15 | 496 |
| 41 | 0.1 | 0.09 | 0.01 | 0.17 | 357 |
| 42 | 0.1 | 0.1 | 0.03 | 0.16 | 262 |
| 43 | 0.11 | 0.11 | 0.05 | 0.17 | 209 |
| 44 | 0.11 | 0.1 | 0.03 | 0.17 | 193 |
| 45 | 0.1 | 0.09 | 0.03 | 0.15 | 215 |
| 46 | 0.08 | 0.08 | 0.02 | 0.14 | 265 |
| 47 | 0.07 | 0.07 | 0.01 | 0.12 | 332 |
| 48 | 0.05 | 0.05 | 0 | 0.11 | 410 |
| 49 | 0.04 | 0.04 | -0.01 | 0.1 | 498 |
| 50 | 0.03 | 0.04 | -0.01 | 0.09 | 596 |
| 51 | 0.03 | 0.03 | -0.02 | 0.08 | 699 |
| 52 | 0.02 | 0.03 | -0.02 | 0.07 | 801 |
| 53 | 0.02 | 0.02 | -0.03 | 0.07 | 900 |
| 54 | 0.02 | 0.02 | -0.03 | 0.07 | 999 |
| 55 | 0.02 | 0.02 | -0.03 | 0.07 | 1091 |
| 56 | 0.02 | 0.02 | -0.02 | 0.06 | 1174 |
| 57 | 0.02 | 0.02 | -0.02 | 0.06 | 1242 |
| 58 | 0.02 | 0.01 | -0.02 | 0.05 | 1300 |
| 59 | 0.02 | 0.02 | -0.02 | 0.06 | 1347 |
| 60 | 0.02 | 0.02 | -0.02 | 0.05 | 1381 |
| 61 | 0.02 | 0.02 | -0.02 | 0.06 | 1401 |
| 62 | 0.03 | 0.03 | -0.01 | 0.06 | 1404 |
| 63 | 0.03 | 0.03 | -0.01 | 0.07 | 1391 |
| 64 | 0.04 | 0.04 | 0 | 0.08 | 1359 |
| 65 | 0.04 | 0.04 | 0 | 0.08 | 1309 |
| 66 | 0.04 | 0.04 | 0 | 0.08 | 1245 |
| 67 | 0.04 | 0.05 | 0.01 | 0.08 | 1169 |
| 68 | 0.04 | 0.04 | 0.01 | 0.08 | 1084 |
| 69 | 0.04 | 0.04 | 0 | 0.09 | 993 |
| 70 | 0.05 | 0.04 | 0 | 0.09 | 897 |
| 71 | 0.05 | 0.04 | 0 | 0.08 | 797 |
| 72 | 0.05 | 0.05 | 0.01 | 0.09 | 695 |
| 73 | 0.05 | 0.04 | -0.01 | 0.09 | 594 |
| 16 | 0.03 | 0.03 | -0.03 | 0.1 | 754 |
| Note: *β*= standardized regression estimate, Bootstrapped *β*= Bootstrapped standardized regression estimate, Bootstrapped 95% CI= empirical 95% confidence intervals, *N*= sum of regression weights. | | | | | |
